# Supplementary material for: Maternal and cohort effects modulate offspring responses to multiple stressors
Source: Proc Biol Sci. 2020 Jun 17;287(1929):20200492. doi: 10.1098/rspb.2020.0492 (PMC7329052; doi:10.1098/rspb.2020.0492)
Supplement: Supplementary figures and tables [file rspb20200492supp1.docx]

**Maternal and cohort effects modulate offspring responses to multiple stressors**

Gabriela Torres, David N. Thomas, Nia M. Whiteley, David Wilcockson, Luis Giménez

Proceedings of the Royal Society B (DOI: 10.1098/rspb.2020.0492)

Figure S1. Experimental design for *Carcinus maenas*. (a) Maternal environment: females (carrying freshly laid eggs) were collected in two seasons (S): Spring-summer and Autumn and were kept under four combinations of two embryonic temperatures (E_T_): 15 and 18ºC, and two embryonic salinities (E_S_): 25 and 35 PSU. (b) Larval environment: after hatching, larvae from each of female were distributed among six combinations of three larval temperatures (L_T_): 15, 18 and 24ºC, and two larval salinities (L_S_): 20 and 35 PSU to determine larval performance (survival and development time to Zoea II).

Table S1. *Carcinus maenas*. Embryonic environment: number of females per treatment in each season. Duration of embryonic development under the chosen treatments.

| Season  (S) | Temperature  (E_T_:°C) | Salinity  (E_S_: PSU) | Number of females | Duration of exposure (days) |
| --- | --- | --- | --- | --- |
| Spring-Summer | 15 | 25 | 3 | 20.0±0.8 |
| Spring-Summer | 15 | 35 | 2 | 18.0±0.7 |
| Spring-Summer | 18 | 25 | 4 | 16.3±0.6 |
| Spring-Summer^*^ | 18 | 35 | 3 | 4,3±0,2 |
| Spring-Summer^*^ | 18 | 35 | 1 | 13 |
| Autumn | 15 | 25 | 3 | 33.3±4.8 |
| Autumn | 15 | 35 | 3 | 39.0±5.7 |
| Autumn | 18 | 25 | 7 | 28.4±1.7 |
| Autumn | 18 | 35 | 6 | 25.5±3.3 |

^*^Note: Due to low number of females with early eggs in the Spring-Summer season, some females with more developed eggs were used. They were exposed for a shorter time (4,3±0,2days) to 18°C and seawater, similar conditions to the natural environment in Spring-summer.

Figure S2. *Carcinus maenas*. Average survival rates to Zoea II, discriminated by larval salinity (L_S_) and temperature (L_T_). Larvae hatched from n (see Table S1) females kept in the laboratory for 4 days (stripped bars) *vs.* larvae hatched from one female kept 13 days (full bars) at optimal conditions (18°C and seawater) during embryonic development. No significant differences were detected between these groups. Values shown as mean ± standard error.

Table S2. *Carcinus maenas*. Survival to Zoea II (logistic transformed data) in response to Season (S), embryonic temperature (E_T_), embryonic salinity (E_S_), larval temperature (L_T_), larval salinity (L_S_) and female of origin (F). Female is a random factor, nested in the interaction S:E_T_:E_S_. The remaining five factors are fixed and form a 5-way factorial design. Model selection on random terms was carried out through REML fitting; since the full model performed considerably better than any alternative model, no test was made and the full random model was retained. Fixed effects were tested after ML fitting. In (a) the table shows the best models within a delta AIC = 10 (model selection of four factorial terms not shown for simplicity). In (b), it shows backward removal of the six interaction terms that were subsequently kept in the best model. The best overall model considers both the best random and fixed structures.

| Model selection: Random (REML) | | AIC |  |  |
| --- | --- | --- | --- | --- |
| Full model: best variance heterogeneity term: L_S_ | |  |  |  |
|  | F:L_T_:L_S_ (best random structure) | 2423 |  |  |
|  | F: L_S_ | 2571 |  |  |
|  | F: L_T_ | 2655 |  |  |
|  | F | 2734 |  |  |
| Model selection: Fixed structure (ML) | |  |  |  |
| (a) Best fixed structure *vs.* larger models | |  |  |  |
|  | Best = 2way **+** interaction terms in (b) | 2387 |  |  |
|  | (1) = Best + E_S_:E_T_:L_T_ | 2387 |  |  |
|  | (2) = (1) + S:E_T_:L_T_ | 2389 |  |  |
|  | (3) = (2) + E_S_:E_T_:L_S_ | 2391 |  |  |
|  | (4) = (3) + S:E_T_:L_S_ | 2391 |  |  |
|  | Full 3-way | 2393 |  |  |
|  | Full 4-way | 2401 |  |  |
|  | Full 5-way (full model) | 2403 |  |  |
| (b) Interaction terms in “Best” | | AIC | LR | P |
|  | S:E_T_:E_S_ | 2390 | 5.56 | 0.018 |
|  | S:E_S_:L_S_ | 2391 | 5.60 | 0.018 |
|  | S:E_S_:L_T_ | 2390 | 7.21 | 0.027 |
|  | S:L_S_:L_T_ | 2392 | 8.73 | 0.013 |
|  | E_S_:L_S_:L_T_ | 2398 | 15.21 | <0.001 |
|  | E_T_:L_S_:L_T_ | 2392 | 8.73 | 0.013 |

Figure S3. *Carcinus maenas*, average percent survival to Zoea II discriminated by larval salinity (L_S_): (a) salinity 20PSU and (b) salinity 35PSU; season (S): Spring-summer cohort (left panels) and autumn cohort (right panels); larval temperature (L_T_): 15, 18 and 24°C (X-axis); female of origin (symbols with connecting lines). Standard error bars were omitted for clarity. Embryonic temperature (E_T_) - lines and symbols: blue (15°C) and green (18°C).

Figure S4. *Carcinus maenas*, survival to Zoea II. (a) Interaction between season (S), embryonic temperature (E_T_) and salinity (E_S_). (b) Interaction between season (S), embryonic salinity (E_S_) and larval temperature (L_T_). In (a) different letters below (or above) symbols denote significant differences between treatments. Values shown as mean ± standard error.

Table S3. *Carcinus maenas*. Duration of development to Zoea II, raw data, in response to Season (S), embryonic temperature (E_T_), embryonic salinity (E_S_), larval temperature (L_T_) and female of origin (F: random factor) when larvae were reared in seawater. Female is a random factor, nested in the interaction S:E_T_:E_S_. The remaining four factors are fixed and form a 4-way factorial design (L_S_: larval salinity is not considered). Model selection on random terms was carried out through REML fitting; since the full model performed considerably better than any alternative model, no test was made and the full random model was retained. Fixed effects were tested after ML fitting. The best overall model considers both the best random and fixed structures.

| Model selection: Random (REML) | | AIC |  |  |
| --- | --- | --- | --- | --- |
|  | F:L_T_ (best random structure) | 973 |  |  |
|  | F | 1016 |  |  |
| Model selection: Fixed (ML) | | AIC | LR | P |
| Best fixed structure *vs.* smaller model | |  |  |  |
|  | Best = 4 way factorial (full model) | 954 | 7.89 | 0.020 |
|  | 3-way factorial | 958 |  |  |

Table S4. *Carcinus maenas*. Duration of development to Zoea II, logarithmic transformed data. Female is a random factor, nested in the interaction S:E_T_:E_S_. The remaining four factors are fixed and form a 4-way factorial design (L_S_: larval salinity is not considered). Model selection on random terms was carried out through REML fitting; since the full model performed considerably better than any alternative model, no test was made and the full random model was retained. Fixed effects were tested after ML fitting. In (a) the table shows the best models within a delta AIC = 10. In (b), it shows backward removal of the six interaction terms that were subsequently kept in the best model. The best model contains non-significant 2-way interaction terms but those were retained as parts of the significant 3-way interaction. The best overall model considers both the best random and fixed structures.

| Model selection: Random (REML) | | AIC |  |  |
| --- | --- | --- | --- | --- |
| Full model: best variance heterogeneity term: L_S_ | |  |  |  |
|  | F:L_T_ (best random structure) | -783 |  |  |
|  | F | -719 |  |  |
| Model selection: Fixed (ML) | |  |  |  |
| (a) Best fixed structure *vs.* larger models | | AIC | LR | P |
|  | Best = Additive+E_S_:E_T_+S:E_S_+S:E_T_+S:L_T_+S:E_S_:E_T_ | -898 |  |  |
|  | Best + E_S_:L_T_ | -896 |  |  |
|  | 2 way + S:E_S_:E_T_ (tested *vs.* 2 way factorial) | -893 | 9.59 | 0.002 |
|  | 2 way + S:E_S_:E_T_ + E_S_:E_T_:L_T_ | -891 |  |  |
|  | 2 way + S:E_S_:E_T_ + E_S_:E_T_:L_T_ + S:E_T_:L_T_ | -889 |  |  |
|  | 3 way factorial | -890 |  |  |
|  | 4 way factorial | -889 |  |  |
|  | 2 way factorial | -886 |  |  |
| (b) Significant interaction terms in “Best” | | AIC | LR | P |
|  | S:E_S_:E_T_ | -890 | 10.33 | 0.006 |
|  | S:L_T_ | -891 | 10.16 | 0.006 |
